# The Rab GTPase-binding protein EHBP1L1 and its interactors CD2AP/CIN85 negatively regulate the length of primary cilia *via* actin remodeling

**DOI:** 10.1016/j.jbc.2023.102985

**Published:** 2023-02-06

**Authors:** Tomohiko Iwano, Tomoaki Sobajima, Sén Takeda, Akihiro Harada, Shin-ichiro Yoshimura

**Affiliations:** 1 Department of Anatomy and Cell Biology, Faculty of Medicine, University of Yamanashi, Chuo, Yamanashi, Japan; 2 Department of Cell Biology, Graduate School of Medicine, Osaka University, Suita, Osaka, Japan; 3 Department of Biochemistry, University of Oxford, Oxford, UK; 4 Department of Anatomy, Teikyo University School of Medicine, Itabashi, Tokyo, Japan

**Keywords:** primary cilium, rab, Arp2/3 complex, actin, cytoskeleton, membrane trafficking, bMERB, bivalent Mical/EHBP Rab binding, C2, Ca^2+^/phospholipid-binding domain, CD2AP, CD2-associated protein, CH, calponin homology, CIN85, Cbl-interacting protein of 85 kDa, CV, ciliary vesicle, DAPI, 4′,6-diamino-2-phenylindole, EGFP, enhanced GFP, EHBP1L1, EH domain-binding protein 1-like 1, ERC, endocytic recycling compartment, FBS, fetal bovine serum, GST, Glutathione S-transferase, hTERT-RPE1, human telomerase reverse transcriptase retinal pigment epithelium 1, PCV, preciliary vesicle, PR, proline-rich region, RPE, retinal pigment epithelium, SH3, SRC homology 3, SR-SIM, super-resolution structured illumination microscopy

## Abstract

Primary cilia are organelles consisting of axonemal microtubules and plasma membranes, and they protrude from the cell surface to the extracellular region and function in signal sensing and transduction. The integrity of cilia, including the length and structure, is associated with signaling functions; however, factors involved in regulating the integrity of cilia have not been fully elucidated. Here, we showed that the Rab GTPase-binding protein EHBP1L1 and its newly identified interactors CD2AP and CIN85, known as adaptor proteins of actin regulators, are involved in ciliary length control. Immunofluorescence microscopy showed that EHBP1L1 and CD2AP/CIN85 are localized to the ciliary sheath. EHBP1L1 depletion caused mislocalization of CD2AP/CIN85, suggesting that CD2AP/CIN85 localization to the ciliary sheath is dependent on EHBP1L1. Additionally, we determined that EHBP1L1- and CD2AP/CIN85-depleted cells had elongated cilia. The aberrantly elongated cilia phenotype and the ciliary localization defect of CD2AP/CIN85 in EHBP1L1-depleted cells were rescued by the expression of WT EHBP1L1, although this was not observed in the CD2AP/CIN85-binding–deficient mutant, indicating that the EHBP1L1–CD2AP/CIN85 interaction is crucial for controlling ciliary length. Furthermore, EHBP1L1- and CD2AP/CIN85-depleted cells exhibited actin nucleation and branching defects around the ciliary base. Taken together, our data demonstrate that the EHBP1L1–CD2AP/CIN85 axis negatively regulates ciliary length *via* actin network remodeling around the basal body.

Primary cilia are antenna-like structures consisting of central axonemal microtubules and membranes. They are exposed to the extracellular environment and function as signaling centers (1, 2). Mutations in ciliary protein-encoding genes, which regulate ciliary length, traffic, and signal transductions, cause a number of disorders termed ciliopathies (3).

Previous studies have described two pathways of ciliogenesis: extracellular and intracellular (2). The extracellular pathway starts with docking of the basal body to the plasma membrane, which occurs mostly in epithelial polarized cells. After docking, the ciliary membrane elongates, accompanied by an axonemal microtubule extension. The intracellular pathway, which is predominant in fibroblasts, neural progenitor cells, and retinal pigment epithelium (RPE) cells, is initiated by the accumulation of preciliary vesicles (PCVs) on the distal appendages of the mother centriole, which is known as the basal body. PCVs fuse together to form a ciliary vesicle (CV). The CV then becomes a nascent ciliary membrane with two distinct domains, ciliary sheath and shaft, surrounding the growing axoneme. Finally, the antenna-like ciliary structure is formed by the fusion of the nascent ciliary membrane to the plasma membrane, followed by membrane and axoneme elongation (4, 5).

Rab GTPases function in various membrane trafficking pathways. GTP-bound Rab proteins recruit specific binding proteins to the membrane and regulate trafficking events such as vesicle fusion, fission, tethering, motility, and membrane remodeling (6).

Rab8 is a member of the Rab family, which is conserved among eukaryotes, and it functions in polarized traffic. In mammalian epithelial cells, Rab8 localizes to post-Golgi vesicles, tubules, or the endocytic recycling compartment (ERC) and regulates exocytic trafficking to the plasma membrane (7, 8). Rab8 also localizes and plays a role in the cilia, which are also regarded as polarized membrane domains (9, 10). However, the precise role that Rab8 plays in cilia remains unclear.

EHBP1L1, a Rab8-binding protein, was previously shown to function in polarized exocytic trafficking in small intestinal epithelial cells (11). EHBP1L1 possesses a Ca^2+^/phospholipid-binding (C2) domain, a calponin homology (CH) domain, a bivalent Mical/EHBP Rab binding (bMERB) domain, and a proline-rich (PR) region, and they bind calcium and phospholipids, actin filaments, Rab8 subfamily proteins (Rab8, 10, 13, and 15), and Bin1/Amphysin2, respectively (12, 13, 14). The Rab8-EHBP1L1-Bin1 axis and Bin1-binding protein dynamin have been proposed to cooperate to generate transport vesicles and tubules from the ERC in epithelial cells (11).

We investigated the involvement of EHBP1L1 in primary cilia integrity, in addition to its role in the ERC and exocytic trafficking in epithelial cells. In this study, we demonstrated that EHBP1L1 and its newly identified binding proteins CD2AP and CIN85 localize to the ciliary sheath membrane, which is a subdomain of the CV, and negatively regulate ciliary length. Our study also showed that depletion of EHBP1L1 and CD2P/CIN85 causes actin nucleation and branching defects around the basal body, suggesting that actin network formation is crucial for maintaining an appropriate length of primary cilia.

## Results

### EHBP1L1 localizes in the ciliary sheath during early primary ciliogenesis and negatively regulates ciliary length

**Figure 1:**
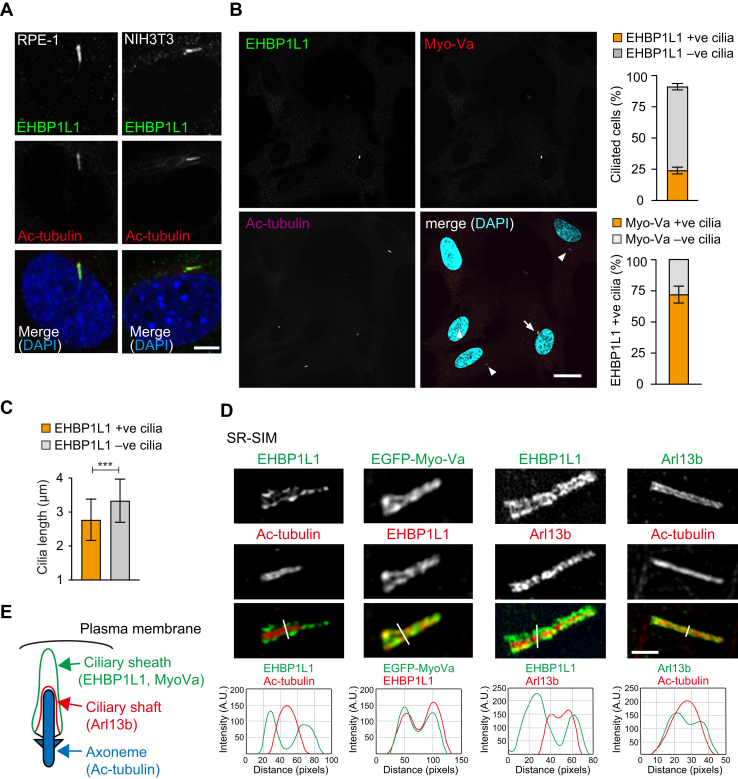
**EHBP1L1 localizes to the ciliary sheath.***A*, hTERT-RPE1 and NIH3T3 cells were immunostained with antibodies against EHBP1L1 (*green*) and acetylated (ac) tubulin (*red*). Nuclei were stained with DAPI (*blue*). Scale bar represents 5 μm. *B*, hTERT-RPE1 cells were immunostained with antibodies against EHBP1L1 (*green*) and myosin-Va (Myo-Va) (*red*), ac-tubulin (*purple*). Nuclei were stained with DAPI (*cyan*). An *arrow* indicates EHBP1L1-positive and Myo-Va–positive cilium. *Arrowheads* indicate only Myo-Va positive cilia. Scale bar represents 20 μm. Percentage of RPE1 cells possessing EHBP1L1-positive (+ve) and EHBP1L1-negative (−ve) cilia, as indicated by ac-tubulin (n = 198 cells) and is indicated on the upper graph. Percentage of (Myo-Va)–positive (+ve) and Myo-Va–negative (−ve) in EHBP1L1 +ve cilia (n = 235 cells) is indicated on the lower graph. *C*, lengths of EHBP1L1 +ve and EHBP1L1 −ve cilia were measured and are indicated on the graph. Error bars represent S.D. *p* values were calculated using Student’s *t* test. Statistical significance was set at ∗∗∗*p* < 0.001. *D*, comparison of ciliary localization of EHBP1L1 with that of ac-tubulin (axoneme), Arl13b (ciliary shaft), and EGFP-Myo-Va (ciliary sheath). Stained cilia were imaged using super-resolution–structured illumination microscopy (SR-SIM). The line scan profiles at positions marked with white lines are also shown. Scale bar represents 1 μm. *E*, graphical summary of the positional relationship between EHBP1L1 and other ciliary proteins. RPE, retinal pigment epithelium.

To determine whether EHBP1L1 is involved in primary cilia formation, two cell lines, hTERT-RPE1 and NIH3T3, whose ciliogenesis is initiated *via* the intracellular pathway, were immunostained with an EHBP1L1 antibody. Conventional confocal microscopy images showed that EHBP1L1 was localized around the primary cilia, as indicated by acetylated tubulin (Fig. 1*A*). Under serum starvation conditions, 91.7% of RPE-1 cells possessed primary cilia while only 24.0% of cilia in ciliated cells were EHBP1L1-positive (Fig. 1*B*). The majority of EHB1P1L1-positive cilia were colabeled with myosin-Va (Myo-Va), which temporarily associates with the nascent ciliary membrane during early ciliogenesis and disappears from the developed cilia in RPE-1 cells (5) (Fig. 1*B*). Moreover, the lengths of EHBP1L1-positive cilia, as indicated by acetylated tubulin, were significantly shorter than those of EHBP1L1-negative cilia (2.77 ± 0.60 μm and 3.34 ± 0.63 μm, respectively) (Fig. 1*C*). Additional analysis was conducted using super-resolution structured illumination microscopy (SR-SIM). SR-SIM analysis revealed that EHBP1L1 localized to the compartment surrounding acetylated tubulin-positive and Arl13b-positive structures that represent the axoneme and the inside of the ciliary membrane called the ciliary shaft membrane during early ciliogenesis, respectively (5) (Fig. 1, *D* and *E*). EHBP1L1 distribution overlapped well with Myo-Va, which was previously shown to localize to the ciliary sheath, which is the outer region of the growing ciliary membrane (5) (Fig. 1, *D* and *E*). These data suggest that EHBP1L1 localizes to the ciliary sheath and functions during early ciliogenesis (Fig. 1*E*).

**Figure 2:**
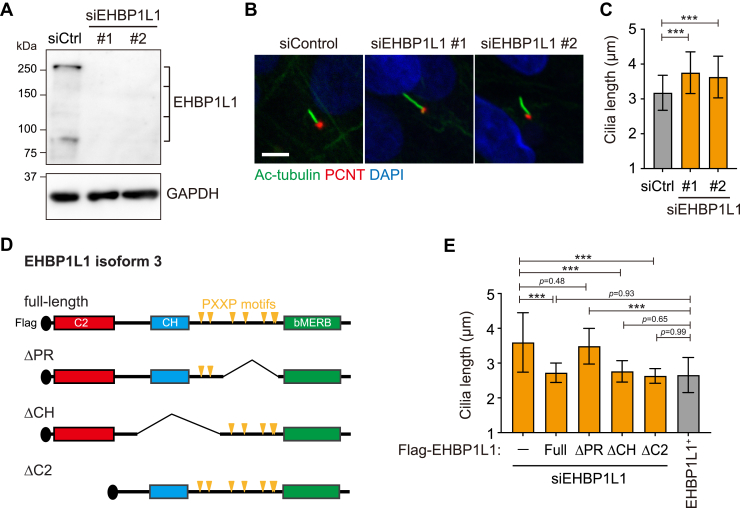
**EHBP1L1 negatively regulates ciliary length.***A*, RPE1 cells were transfected with control (Ctrl) or two EHBP1L1 siRNAs (#1 and #2). EHBP1L1 protein depletion was analyzed by immunoblotting using an EHBP1L1 antibody. GAPDH was used as the loading control. *B*, cells treated with control or EHBP1L1 siRNAs (#1 and #2) were immunostained with ac-tubulin (*green*) and pericentrin (PCNT) (*red*) to visualize primary cilia and basal bodies, respectively. Scale bar represents 5 μm. *C*, ciliary lengths, as indicated by ac-tubulin in control or EHBP1L1 siRNA-transfected cells (#1 and #2), were measured and are indicated on the graph (n = 241–277 cells). Error bars represent the S.D. *p* values were calculated using ordinary one-way ANOVA followed by Tukey’s test. Statistical significance was set at ∗∗∗*p* < 0.001. *D*, schematic drawing of full-length FLAG-tagged EHBP1L1 and deletion mutants used for the rescue experiment. *Orange triangles* indicate the proline-X-X-proline (PXXP) motif. *E*, EHBP1L1-depleted cells (siEHBP1L1) were expressed with or without (−) FLAG-tagged full-length EHBP1L1 and deletion mutants (Full, ΔPR, ΔCH, and ΔC2). Flag-positive ciliary length was measured and is indicated on the graph. The length of endogenous EHBP1L1-positive cilia in control cells (EHBP1L1^+^) is shown for comparison. Ciliary length was measured using Arl13b signal (n = 72–159 cells). Error bars represent the S.D. *p* values were calculated using ordinary one-way ANOVA followed by Tukey’s test. Statistical significance was set at ∗∗∗*p* < 0.001. bMERB, bivalent Mical/EHBP Rab binding domain; C2, Ca^2+^/phospholipid-binding domain; CH, calponin homology domain; PR, proline-rich region; RPE, retinal pigment epithelium.

Next, we determined whether EHBP1L1 plays a role in primary ciliogenesis. To do this, we depleted EHBP1L1 protein using two independent siRNA oligonucleotides (Fig. 2*A*). Compared with the length of primary cilia in control cells (3.18 ± 0.50 μm), EHBP1L1-depleted cells had longer cilia (siRNA #1:3.76 ± 0.59 μm; siRNA #2:3.63 ± 0.60 μm) (Fig. 2, *B* and *C*). These data suggest that EHBP1L1 is involved in the shortening of primary cilia. To identify the region in EHBP1L1 that is involved in the regulation of primary ciliary length, a series of Flag-tagged deletion mutants (Figs. 2*D* and S1) were expressed in EHBP1L1-depleted cells, and phenotypic rescue of ciliary length was assessed. Of the four proteins targeted to the ciliary region (EHBP1L1 full-length, ΔPR, ΔCH, and ΔC2) (Fig. S1), only ΔPR-positive cilia were longer than the endogenous EHBP1L1-positive cilia in control cells (2.66 ± 0.45 μm), and their lengths were similar to those of cilia in EHBP1L1-depleted cells (3.49 ± 0.51 μm and 3.59 ± 0.85 μm, respectively) (Fig. 2*E*). These data indicate that the ΔPR mutant failed to rescue the ciliary phenotype (length) and suggest that the PR region of EHBP1L1 is necessary for the control of ciliary length.

We also demonstrated that the EHBP1L1 mutant lacking the bMERB domain (ΔbMERB) necessary for GTP-Rab8 binding failed to localize to CVs (Fig. S1). Thus, these data clearly indicate the importance of Rab8 in ciliary targeting of EHBP1L1-CD2AP/CIN85.

### CD2AP/CIN85 proteins interact with EHBP1L1

**Figure 3:**
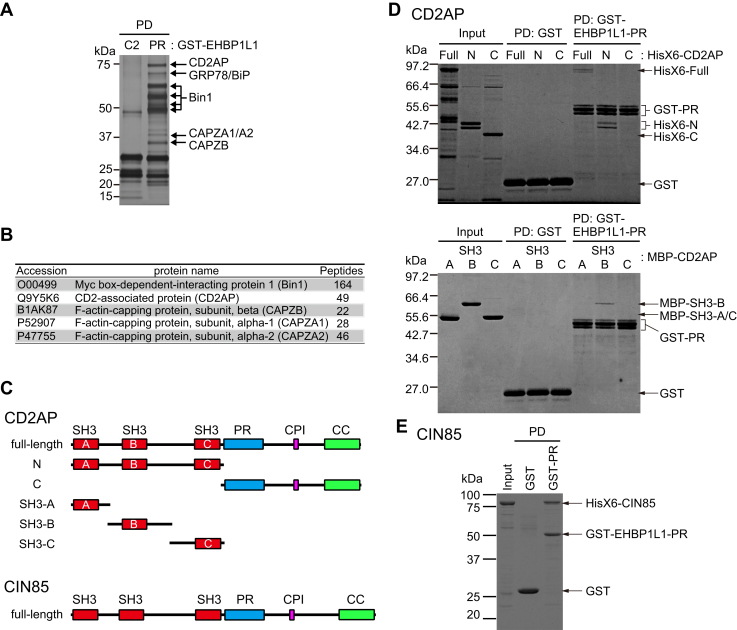
**EHBP1L1 binds CD2AP family proteins.***A*, cell lysate was incubated with immobilized GST-C2 or PR (442–595 a.a.) region of EHBP1L1. Isolated proteins were analyzed using SDS-PAGE and silver staining. *Arrows* indicate specific proteins that coprecipitated with GST-PR but not GST-C2. *B*, summary of proteins identified using mass spectrometry. *C*, schematic drawing of domain structures in the CD2AP protein and its deletion forms (full-length, N and C) and CIN85. *D*, GST-pull-down (PD) assay using purified recombinant proteins. Hisx6-tagged full-length CD2AP, its deletion forms (Full, N, and C), MBP-tagged SH3 domains of CD2AP (SH3-A, -B, and -C), and GST-tagged PR region of EHBP1L1. GST protein was used as a control. The gels were stained with Coomassie brilliant blue (CBB). *E*, GST PD assay using GST or GST-PR and Hisx6-tagged CIN85. CC, coiled-coil domain; CPI, capping protein–interaction motif; GST, Glutathione S-transferase. PR, proline-rich region; SH3, Src-homology 3 domain.

To gain further insights into the function of EHBP1L1 in regulating ciliary length, we attempted to identify the protein that binds to its PR region. Glutathione S-transferase (GST) pull-down assay using the PR region of EHBP1L1 generated several coprecipitated proteins that were absent in the assay conducted using the C2 domain (Fig. 3*A*). Mass spectrometry analysis revealed that Bin1, CD2AP, CAPZA1, CAPZA2, and CAPZB were coprecipitated with the PR region of EHBP1L1 (Fig. 3*B*). We previously reported that the EHBP1L1-Bin1-dynamin axis regulates exocytic trafficking in the endocytic recycling compartment (11). However, the relationship between EHBP1L1, an adaptor for the actin network regulator CD2AP, and the barbed-end actin capping protein CAPZ has not been explored. Thus, we focused on CD2AP and CAPZ proteins.

Given that CAPZ proteins reportedly bind CD2AP and its paralog CIN85 *via* capping protein interaction motifs (15, 16) and CD2AP/CIN85 have three SRC homology 3 (SH3) domains (SH3-A, SH3-B, and SH3-C) (Fig. 3*C*) that are known to bind to PR sequences (17, 18), the CD2AP–CAPZ complex was co-pulled down with the EHBP1L1 PR region *via* the SH3 domain in CD2AP. We examined the direct interaction between CD2AP and the EHBP1L1 PR region using the GST pull-down assay. The results clearly showed direct binding of the full-length and N-terminal regions of CD2AP to GST-EHBP1L1-PR (Fig. 3, *C* and *D*). Further experiments demonstrated that the second SH3 domain (SH3-B) interacted directly with GST-EHBP1L1-PR (Fig. 3*D*). We also confirmed direct binding between the PR region of EHBP1L1 and CIN85 (Fig. 3, *C* and *E*).

### Interaction between EHBP1L1 and CD2AP/CIN85 is crucial for EHBP1L1’s regulation of ciliary length

**Figure 4:**
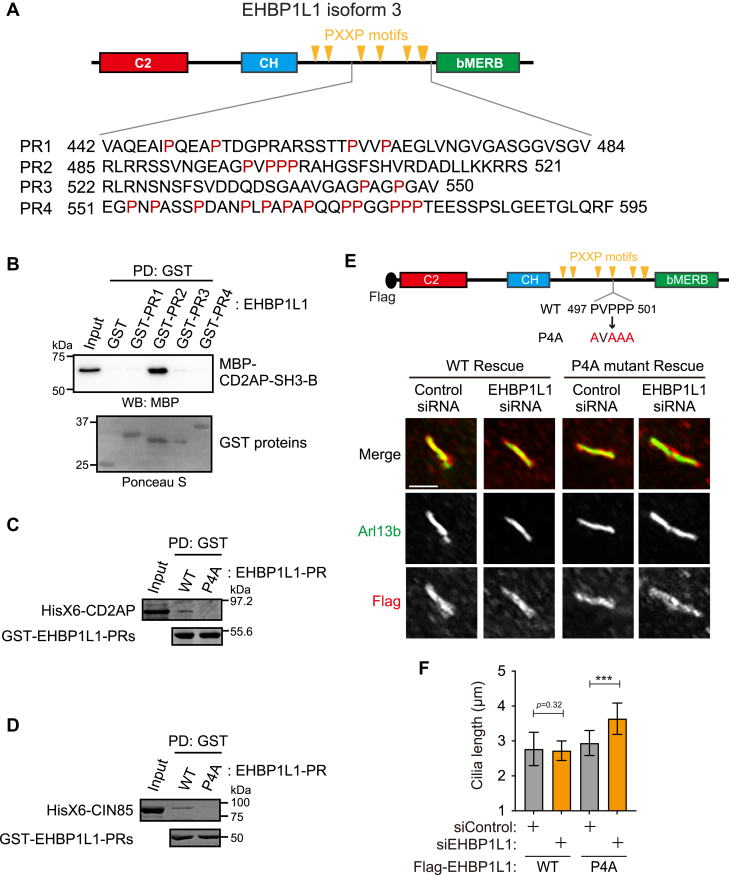
**Interaction between EHBP1L1 and CD2AP/CIN85 protein is crucial for EHBP1L1’s regulation of ciliary length.***A*, amino acid sequences of different fragments of PR regions (PR1-PR4) of EHBP1L1 protein. *B*, interaction between GST-PR1-PR4 and the MBP- SH3-B domain of CD2AP was examined. The pulled-down proteins were immunoblotted using an MBP antibody. Ponceau S–stained membranes showed GST-fused proteins. *C* and *D*, GST pull-down experiment using GST-PR (EHBP1L1_442–595_) WT and P4A mutant and Hisx6-tagged CD2AP (*C*) or CIN85 (*D*). Protein gels were stained with CBB. *E* and *F*, flag-tagged EHBP1L1 full-length WT or P4A mutant were expressed in cells treated with control or EHBP1L1 #1 siRNA. The cells were stained with Flag and Arl13b antibodies (*E*). Scale bar represents 2 μm. Cilia length, indicated using Arl13b, was measured and is indicated on the graph (*F*) (n = 78–134 cells). Error bars represent S.D. *p* values were calculated using ordinary one-way ANOVA followed by Tukey’s test. Statistical significance was set at ∗∗∗*p* < 0.001. GST, Glutathione S-transferase; PR, proline-rich region.

To determine the minimal EHBP1L1 PR region necessary for binding to CD2AP/CIN85, the PR region of EHBP1L1 was divided into four fragments, each containing several SH3-binding proline-X-X-proline (PXXP) motifs (Fig. 4*A*). GST pull-down assays of these fragments bound to CD2AP-SH3-B revealed that the second PR region fragment (PR2) was required for binding to CD2AP-SH3-B (Fig. 4*B*). We further examined the interaction between full-length CD2AP and CIN85 and either the PR region of EHBP1L1 (residues 442–595) or the mutant EHBP1L1-PR in which all four proline residues (positions 497 and 499–501) were replaced with alanine (EHBP1L1-PR P4A). Consistent with the data shown in Figure 3, *D* and *E*, CD2AP and CIN85 bound the WT GST-PR, but not the mutant GST-PR P4A (Fig. 4, *C* and *D*). These data indicate that proline residues 497 to 501 in EHBP1L1 are important for binding to CD2AP and CIN85. Next, we examined the expression of the full-length EHBP1L1 and the EHBP1L1-P4A mutant (Fig. 4, *E* and *F*) in control and EHBP1L1 siRNA-treated cells. Protein expression was confirmed using immunoblotting (Fig. S1). Both WT and P4A mutant were localized around the cilia (Fig. S1). In control siRNA-treated cells, neither WT nor P4A mutant expression affected ciliary length (Fig. 4, *E* and *F*). In EHBP1L1 siRNA-treated cells, elongated cilia (also shown in Fig. 2) was suppressed by the expression of the WT protein but not the P4A mutant (Fig. 4, *E* and *F*). These data suggest that the interaction between EHBP1L1 and the CD2AP/CIN85 proteins is crucial for EHBP1L1’s ability to maintain a certain length for primary cilia.

### CD2AP/CIN85 proteins regulate ciliary length by remodeling the actin network

**Figure 5:**
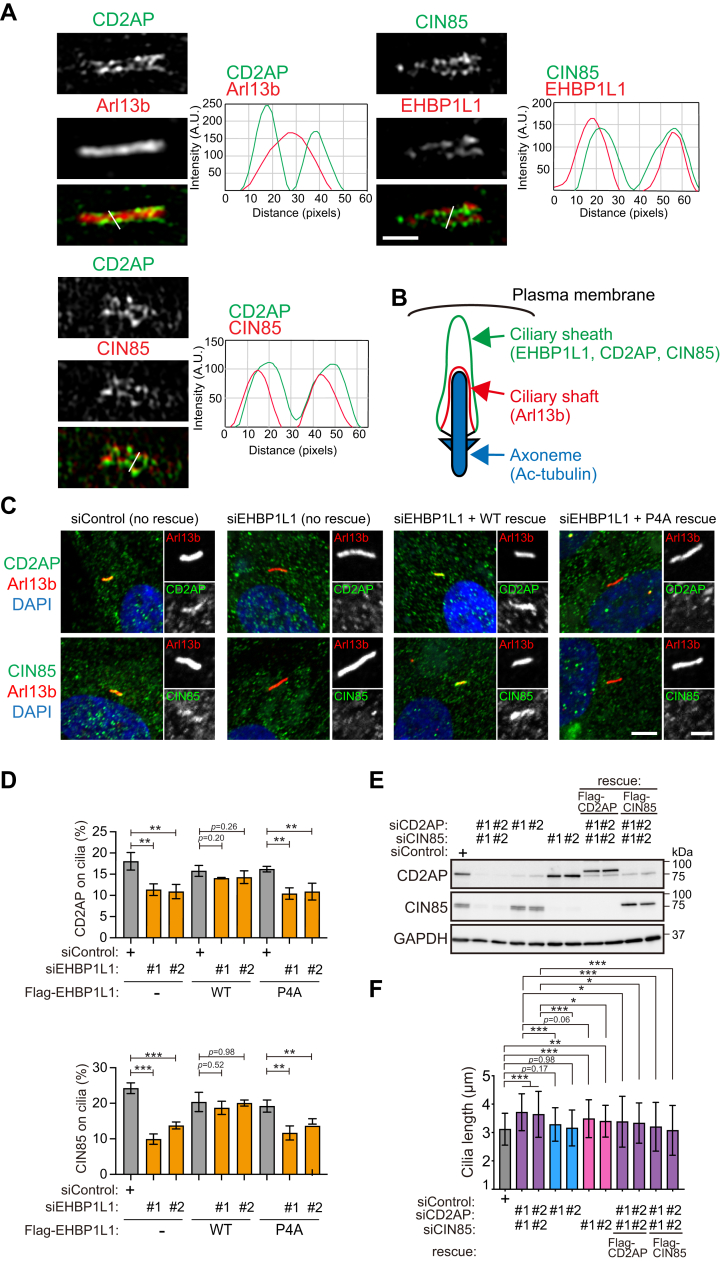
**EHBP1L1-CD2AP/CIN85 axis controls ciliary length.***A*, comparison of ciliary localization of CD2AP and CIN85 to Arl13b (ciliary shaft) and EHBP1L1 (ciliary sheath). The stained cells were imaged using super-resolution structured illumination microscopy (SR-SIM). The line scan profiles at positions marked with *white lines* are also shown. Scale bar represents 1 μm. *B*, graphical summary of the positional relationship between EHBP1L1 and other ciliary proteins. *C*, EHBP1L1-depleted cells and EHBP1L1-depleted cells expressing WT or P4A mutant EHBP1L1 were stained with antibodies against CD2AP, CIN85 (*green*), and Arl13b (*red*). Enlarged pictures of cilia are shown on the right. Scale bar represents (main images) 5 μm; (insets) 2 μm. *D*, percentages of ciliary localization of CD2AP (*upper*) and CIN85 (*lower*) in control, EHBP1L1-depleted, and EHBP1L1-depleted cells expressing WT or P4A mutant were indicated on the graph. *n* = 3 experiments. *E*, the depletion efficiencies and exogenous expression of CD2AP and CIN85 were examined by immunoblotting using control or specific siRNA (#1 and #2)-transfected cells. GAPDH was used as the loading control. *F*, ciliary length indicated by ac-tubulin was measured and is indicated on the graph (n = 89–128 cells). Error bars represent the S.D. *p* values were calculated using ordinary one-way ANOVA followed by Tukey’s test. Statistical significance was set at ∗*p* < 0.05, ∗∗*p* < 0.01, and ∗∗∗*p* < 0.001.

To further assess the relationship between CD2AP/CIN85 and EHBP1L1, cells were costained with antibodies against CD2AP, CIN85, Arl13b, and EHBP1L1 and observed using SR-SIM. CD2AP was localized to the same region as CIN85, which was distinguishable from the Arl13b-positive ciliary shaft, whereas CIN85 overlapped with EHBP1L1 (Fig. 5*A*). These data indicate that CD2AP/CIN85 localized to the ciliary sheath together with EHBP1L1 (Fig. 5*B*). The number of CD2AP- and CIN85-positive cilia was significantly lower in EHBP1L1-depleted cells. Ciliary localization defects in CD2AP and CIN85 in EHBP1L1-depleted cells were rescued by the expression of WT EHBP1L1 but not by the expression of the EHBP1L1-P4A mutant (Fig. 5, *C* and *D*). These results suggest that recruitment of CD2AP/CIN85 to the ciliary sheath is dependent on EHBP1L1. Next, we investigated the role of CD2AP/CIN85 in the determination of ciliary length using siRNA (Fig. 5*E*). Compared with control cells, CD2AP/CIN85-depleted cells had longer cilia, and this trait was suppressed by exogenous expression of either CD2AP or CIN85 (Fig. 5*F*). These data indicate that the EHBP1L1–CD2AP/CIN85 axis controls ciliary length.

**Figure 6:**
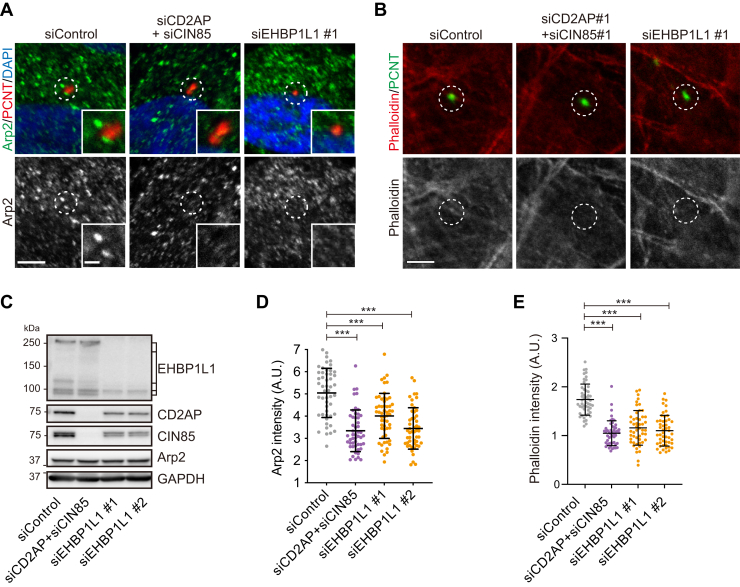
**CD2AP/CIN85 promotes actin-branched network formation around the base of primary cilia.***A* and *B*, RPE1 cells transfected with EHBP1L1 #1 siRNA or cotransfected with CD2AP and CIN85 siRNAs were stained with antibodies against Arp2 (*A*) and PCNT (*A* and *B*) or with Alexa-568 phalloidin (*B*). *Dotted circles* indicate a 1 μm radius around the PCNT-labeled basal body. Magnified images of the *circled areas* are shown in the *insets*. Scale bar represent (main images) 2 μm; (insets) 0.5 μm. *D* and *E*, fluorescence intensities of Arp2 and Alexa-568 phalloidin within a 1 μm radius around the basal body were measured and plotted against siRNA-treated cells (control, CD2AP/CIN85, or EHBP1L1 #1 and #2). n = 50 for each experiment. Error bars represent S.D. *p* values were calculated using Student’s *t* test. Statistical significance was set at ∗∗∗*p* < 0.001. *C*, cell lysates treated with siRNAs (control, CD2AP/CIN85, or EHBP1L1 #1 and #2) were immunoblotted with EHBP1L1, CD2AP, CIN85, and Arp2 antibodies. GAPDH was used as the loading control. PCNT, pericentrin; RPE, retinal pigment epithelium.

Since CD2AP family proteins and the CAPZ complex regulate actin network formation by enhancing actin branching (19) and actin nucleation at the centrosome/basal body in an Arp2/Arp3-dependent manner (20), we examined the distribution of Arp2 to monitor the actin-branched network around the basal body in EHBP1L1- and CD2AP/CIN85-depleted cells. We observed significantly reduced Arp2 fluorescence intensity around the basal body (Fig. 6, *A* and *D*), although the quantity of Arp2 protein did not change (Fig. 6*C*). In addition, we also observed that fluorescent-dye–conjugated phalloidin-labeled actin filaments around the basal body in EHBP1L1- and CD2AP/CIN85-depleted cells had reduced signal intensity (Fig. 6, *B* and *E*). Taken together, these results suggest that EHBP1L1-CD2AP/CIN85-CAPZ–mediated actin-branched network formation around the basal body controls ciliary length.

## Discussion

The lengths of primary cilia are often aberrant in cells carrying mutated ciliopathy-associated genes (21, 22, 23, 24, 25, 26, 27, 28, 29, 30, 31), suggesting that tight regulation of ciliary length is crucial for ciliary functions. The maintenance of ciliary length is dependent on the integrity of the ciliary membrane and axonemal microtubules (32, 33). In addition, several lines of evidence have indicated that actin plays an important role in controlling ciliary length. Cells treated with drugs or siRNAs targeting actin-regulating genes that inhibit actin polymerization showed an increased ciliated cell number as well as elongated cilia (34, 35, 36).

Although the precise role of actin in ciliogenesis is unclear, several mechanisms have been proposed. For instance, the actin network around the centrosome/basal body may inhibit the vesicle trafficking necessary to form CVs or elongate the ciliary membrane (37). Given the association between actin filaments and the basal body (20), it is likely that the actin network determines the position of the basal body close to the plasma membrane, a step that is important for ciliogenesis (4).

CD2AP and the paralog CIN85 are adaptor proteins that interact with the membrane or signaling proteins *via* the SH3 domain (18) and with barbed-end actin capping proteins (CAPZs) *via* the capping protein interaction motif (16). Importantly, previous studies demonstrated that the C-termini of CD2AP family proteins have the ability to uncap CAPZ from actin filaments and subsequently polymerize actin (38, 39). Moreover, CD2AP associates with and stabilizes actin filaments and bundles (40, 41). In this study, we demonstrated that CD2AP/CIN85 is recruited by EHBP1L1 to the ciliary sheath, a subdomain of the CV, *via* the SH3 domain (Figs. 1 and 5). Our study also showed that depletion of EHBP1L1 and CD2AP/CIN85 causes the actin filaments around the basal body to become unstable, as indicated by the absence of the Arp2/3 complex (Fig. 6). Thus, our findings strongly suggest that CD2AP/CIN85 functions as an adaptor that links CV to actin filaments and an actin regulator that stabilizes and develops the actin network, thereby negatively regulating primary ciliogenesis. Notably, the CH domain in EHBP1L1 can bind directly to actin (42, 43). Despite the nonessential role of the CH domain in the control of ciliary length (Fig. 2, *D* and *E*), we hypothesize that the direct interaction between EHBP1L1 and actin may support the role of CD2AP/CIN85 in actin network formation.

Previous studies have demonstrated that Rab8 is a positive regulator of primary cilia formation that is involved in PCV assembly during early ciliogenesis and vesicle trafficking to the ciliary base *via* various binding proteins that interact with the membrane, centrosome/basal body, and cytoskeleton; however, whether these proteins bind directly to Rab8 has not been determined (9, 10, 44, 45, 46, 47, 48). In summary, overexpression of the WT or GTP-restricted form of Rab8 promotes ciliogenesis and leads to extensive cilia. In contrast, depletion of Rab8 and various Rab8-binding proteins completely inhibits ciliary formation or reduces ciliary length. Contrary to the cited studies, we revealed that EHBP1L1-CD2AP/CIN85 has a negative effect on ciliary length, as indicated by the elongated cilia in EHBP1L1- and CD2AP/CIN85-depleted cells. These findings suggest that the role of Rab8 in ciliogenesis and ciliary integrity is unexpectedly complex.

In this study, we discovered a novel mechanism for primary ciliogenesis involving Rab8. Our findings suggest that Rab8 plays an indispensable role in ciliary membrane trafficking and actin network formation *via* EHBP1L1-CD2AP/CIN85, thus supporting the concept that tight regulation of the actin network is crucial for controlling ciliary length.

## Experimental procedures

### Antibodies

Rabbit and rat polyclonal anti-EHBP1L1 antibodies were raised as previously described (11). The following additional primary antibodies were used: rabbit anti-pericentrin (ATLAS antibodies, #HPA016820); mouse anti-acetylated tubulin and anti-FLAG M2 (Sigma-Aldrich, #T7451 and #F1804); mouse anti-Arl13b (Abcam, #ab136648); rabbit anti-ß-actin, anti-Arl13b, anti-CD2AP, and mouse anti-GAPDH (Proteintech, #20536-1-AP, #17711-1-AP, #51046-1-AP, and #66004-1-Ig); mouse anti-CIN85 and anti-Arp2 (Santa Cruz Biotechnology, #sc-166862 and #sc-166103); rabbit anti-myosin Va (Cell signaling, #3402) and mouse anti-MBP (Developmental Studies Hybridoma Bank). The secondary antibodies Alexa Fluor 488 donkey anti-rabbit IgG, anti-mouse IgG, Alexa Fluor 568 donkey anti-rabbit IgG, and anti-mouse IgG were purchased from Thermo Fisher Scientific. Horseradish peroxidase–conjugated anti-rabbit IgG and anti-mouse IgG were purchased from Cell Signaling Technology.

### Cell culture

Human telomerase-immortalized retinal pigmented epithelial cells (hTERT-RPE1) were cultured in a 1:1 mixture of Dulbecco’s modified Eagle’s medium and Ham’s F-12 medium (FUJIFILM Wako), supplemented with 2 mM L-glutamine (FUJIFILM Wako), 10% fetal bovine serum (FBS, Thermo Fisher Scientific), and 0.375% sodium bicarbonate. NIH3T3 cells were cultured in Dulbecco’s modified Eagle’s medium supplemented with 10% FBS. All cells were incubated at 37 °C in a humidified 5% CO_2_/95% air atmosphere.

### Plasmid construction

C-terminus (1148–1855 a.a.) of *Homo sapiens* myosin-Va cDNA was amplified by PCR using KOD -Plus- Neo (TOYOBO) and subcloned into the pEGFP-C3 vector. FLAG-tagged constructs encoding EHBP1L1 (11) were transferred into the lentiviral vector pCSII-CMV-MCS-IRES2-Bsd (RIKEN BRC, #RDB04385). *Mus musculus* CD2AP and *H. sapiens* CIN85 cDNAs amplified by PCR from mouse 17-day embryo and human fetus Marathon Ready cDNA (Takara Bio, Clontech) were subcloned into pCSII-CMV-FLAG-MCS-IRES2-Bsd to express N-terminally FLAG-tagging proteins. The siRNA-resistant mutants of EHBP1L1, CD2AP, and CIN85 used for the rescue experiments were generated through site-directed mutagenesis. The target sequence was changed as follows: 5′-cgattctacccagacaaga-3′ to 5′-cgGttTtaTccTgaTaaga-3′ (EHBP1L1), 5′-ctggagcagtgtacccaaag-3′ to 5′-ctggagcCgtAtaGccGaag-3′ (CD2AP), and 5′-gactgttaccatatcccaa-3′ to 5′-gacCgttAacAatTtcGcaa-3′ and 5′-ggaGcgAagTaaCgaTaat-3′ (CIN85). For protein expression and purification, full-length and deletion mutants of CD2AP and CIN85 were inserted into a pQE32-TEV vector to produce Hisx6-tagged proteins. The EHBP1L1-PR region was inserted into the pFAT2 vector to produce Hisx6-GST–tagged protein. CD2AP deletion constructs were inserted into the pMAL-pre-His vector to produce Hisx6-MBP–tagged proteins.

### Production and infection of lentivirus

Lenti-X 293T cells (Takara) were cotransfected with pCSII plasmids, pCMV-VSV-G-RSV-Rev (RIKEN BRC #RDB04393), and pCAG-HIVgp (RIKEN BRC #RDB04394). The medium containing the lentiviruses was collected and filtered through a 0.45 μm pore-size membrane filter 72 h after transfection. hTERT-RPE1 cells were combined with a 1:10 lentivirus solution and the cells selected using 20 μg/ml blasticidin S (FUJIFILM Wako).

### Protein expression and purification

Bacterial expression plasmids were transformed into Rosetta 2 (DE3) pLys cells (Novagen) and incubated at 18 °C for 16 h in the presence of 0.25 mM IPTG. The cell lysates were incubated with Ni-NTA agarose (Qiagen) for 2 h and bound proteins were eluted with 200 mM imidazole. The proteins were dialyzed against PBS, snap-frozen with liquid N_2_, and stored at −80 °C until use.

### Glutathione S-transferase pull-down and mass spectrometry

GST pull-down of purified proteins and pull-down using the cell lysate were performed as previously described (11). Protein samples were separated on NuPAGE Novex Bis-Tris gels (Thermo Fisher Scientific) and then silver stained as previously described (49). The bands were excised from the gel, and the proteins reduced, alkylated, and digested with trypsin in Tris-buffered saline for 16 h at 37 °C. The proteins were analyzed using Q-Exactive mass spectroscopy (Thermo Fisher Scientific) at the Osaka University Center for Medical Research and Education. Searches were conducted against the Mascot server (v2.3; Matrix Science) and International Protein Index (mouse, Ver. 3.77 or 3.87; EMBL-EBI) databases.

### Small interfering RNA

siRNAs for EHBP1L1 (Sigma-Aldrich siRNA SASI_Hs02_00320623, Qiagen siRNA #SI00377055), CD2AP (Ambion Silencer select #s24191 and #s24193, Thermo Fisher Scientific), and CIN85 (Ambion Silencer select #s26895 and #s26897, Thermo Fisher Scientific), as well as a negative control (Ambion Silencer select negative control No. 1 #4390843, Thermo Fisher Scientific) were used.

### Transfection and immunofluorescence

To analyze cilia, 0.5 × 10^5^ cells were cultured on coverslips in 24-well plates. To express EGFP-Myo-Va, 0.5 μg of pEGFP-C3-Myo-Va plasmid was transfected using Viafect reagent (Promega) in medium containing 10% FBS. After 48 h, the medium was replaced with serum-free medium, and the cells were incubated for a further 24 h. For knockdown experiments, 5 pmol of siRNA was transfected using Lipofectamine RNAiMax reagent (Thermo Fisher Scientific) in medium containing 10% FBS. After 48 h, siRNA was transfected again in serum-free medium, and cells were incubated for a further 72 h. Cells were then fixed with 3% paraformaldehyde in PBS for 10 min and washed twice with 50 mM NH_4_Cl in PBS and twice with PBS. For Arp2 and pericentrin staining, cells were fixed with methanol and acetone (1:1) for 20 min at −20 °C and then rinsed with PBS. The cells were then incubated with primary and secondary antibodies in the presence of 0.1% saponin. To visualize F-actin, paraformaldehyde-fixed and saponin-permeabilized cells were incubated with Alexa-568 phalloidin (Thermo Fisher Scientific) for 60 min and then rinsed with PBS. The coverslips were mounted with ProLong Diamond Antifade Mountant containing DAPI (Thermo Fisher Scientific) and observed using Olympus IX71 and Nikon A1R HD25 confocal microscopes. The lengths of cilia indicated by acetylated tubulin or Arl13b staining were measured using ImageJ and analyzed using Microsoft Excel and GraphPad Prism statistical software. Images were processed using Adobe Photoshop.

### Super-resolution structured illumination microscopy

Samples were imaged using an ELYRA S.1 microscope with a 100×/1.46 oil-immersion objective (Carl Zeiss). Images and fluorescence intensity profiles were analyzed using ZEN 2011 software (Carl Zeiss) and image J.

## Data availability

All the data analyzed in this study are included in the main article and supporting information.

## Supporting information

This article contains supporting information.

## Conflict of interest

The authors declare that they have no competing interests.

## References

[1] Malicki J.J., Johnson C.A. (2017). The cilium: cellular antenna and central processing unit. Trends Cell Biol..

[2] Bernabé-Rubio M., Alonso M.A. (2017). Routes and machinery of primary cilium biogenesis. Cell. Mol. Life Sci..

[3] Anvarian Z., Mykytyn K., Mukhopadhyay S., Pedersen L.B., Christensen S.T. (2019). Cellular signalling by primary cilia in development, organ function and disease. Nat. Rev. Nephrol..

[4] Sorokin S.P. (1968). Reconstruction of centriole formation and ciliogenesis in mammalian lungs. J. Cell Sci..

[5] Wu C.T., Chen H.Y., Tang T.K. (2018). Myosin-Va is required for preciliary vesicle transportation to the mother centriole during ciliogenesis. Nat. Cell Biol..

[6] Hutagalung A.H., Novick P.J. (2011). Role of Rab GTPases in membrane traffic and cell physiology. Physiol. Rev..

[7] Ang A.L., Fölsch H., Koivisto U.M., Pypaert M., Mellman I. (2003). The Rab8 GTPase selectively regulates AP-1B-dependent basolateral transport in polarized Madin-Darby canine kidney cells. J. Cell Biol..

[8] Sato T., Mushiake S., Kato Y., Sato K., Sato M., Takeda N. (2007). The Rab8 GTPase regulates apical protein localization in intestinal cells. Nature.

[9] Nachury M.V., Loktev A.V., Zhang Q., Westlake C.J., Peränen J., Merdes A. (2007). A Core complex of BBS proteins cooperates with the GTPase Rab8 to promote ciliary membrane biogenesis. Cell.

[10] Yoshimura S., Egerer J., Fuchs E., Haas A.K., Barr F.A. (2007). Functional dissection of Rab GTPases involved in primary cilium formation. J. Cell Biol..

[11] Nakajo A., Yoshimura S., Togawa H., Kunii M., Iwano T., Izumi A. (2016). EHBP1L1 coordinates Rab8 and Bin1 to regulate apical-directed transport in polarized epithelial cells. J. Cell Biol..

[12] Davletov B.A., Sudhof T.C. (1993). A single C2 domain from synaptotagmin I is sufficient for high affinity Ca2+/phospholipid binding. J. Biol. Chem..

[13] Korenbaum E., Rivero F. (2002). Calponin homology domains at a glance. J. Cell Sci..

[14] Rai A., Oprisko A., Campos J., Fu Y., Friese T., Itzen A. (2016). bMERB domains are bivalent Rab8 family effectors evolved by gene duplication. Elife.

[15] Hutchings N.J., Clarkson N., Chalkley R., Barclay A.N., Brown M.H. (2003). Linking the T cell surface protein CD2 to the actin-capping protein CAPZ via CMS and CIN85. J. Biol. Chem..

[16] Edwards M., Zwolak A., Schafer D.A., Sept D., Dominguez R., Cooper J.A. (2014). Capping protein regulators fine-tune actin assembly dynamics. Nat. Rev. Mol. Cell Biol..

[17] Dustin M.L., Olszowy M.W., Holdorf A.D., Li J., Bromley S., Desai N. (1998). A novel adaptor protein orchestrates receptor patterning and cytoskeletal polarity in T-cell contacts. Cell.

[18] Moncalián G., Cárdenes N., Deribe Y.L., Spínola-Amilibia M., Dikic I., Bravo J. (2006). Atypical polyproline recognition by the CMS N-terminal Src homology 3 domain. J. Biol. Chem..

[19] Zhao J., Bruck S., Cemerski S., Zhang L., Butler B., Dani A. (2013). CD2AP links cortactin and capping protein at the cell periphery to facilitate formation of lamellipodia. Mol. Cell. Biol..

[20] Farina F., Gaillard J., Guérin C., Couté Y., Sillibourne J., Blanchoin L. (2016). The centrosome is an actin-organizing centre. Nat. Cell Biol..

[21] Putoux A., Thomas S., Coene K.L.M., Davis E.E., Alanay Y., Ogur G. (2011). KIF7 mutations cause fetal hydrolethalus and acrocallosal syndromes. Nat. Genet..

[22] Sanders A.A.W.M., de Vrieze E., Alazami A.M., Alzahrani F., Malarkey E.B., Sorusch N. (2015). KIAA0556 is a novel ciliary basal body component mutated in Joubert syndrome. Genome Biol..

[23] Doornbos C., van Beek R., Bongers E.M.H.F., Lugtenberg D., Klaren P.H.M., Vissers L.E.L.M. (2021). Cell-based assay for ciliopathy patients to improve accurate diagnosis using ALPACA. Eur. J. Hum. Genet..

[24] Gerhardt C., Lier J.M., Burmüh S., Struchtrup A., Deutschmann K., Vetter M. (2015). The transition zone protein Rpgrip 1l regulates proteasomal activity at the primary cilium. J. Cell Biol..

[25] Taylor S.P., Dantas T.J., Duran I., Wu S., Lachman R.S., Nelson S.F. (2015). Mutations in DYNC2LI1 disrupt cilia function and cause short rib polydactyly syndrome. Nat. Commun..

[26] Lambacher N.J., Bruel A.L., Van Dam T.J.P., Szymaska K., Slaats G.G., Kuhns S. (2016). TMEM107 recruits ciliopathy proteins to subdomains of the ciliary transition zone and causes Joubert syndrome. Nat. Cell Biol..

[27] Grampa V., Delous M., Zaidan M., Odye G., Thomas S., Elkhartoufi N. (2016). Novel NEK8 mutations cause severe syndromic renal cystic dysplasia through YAP dysregulation. PLoS Genet..

[28] Airik R., Schueler M., Airik M., Cho J., Ulanowicz K.A., Porath J.D. (2016). SDCCAG8 interacts with rab effector proteins rabep2 and erc1 and is required for Hedgehog signaling. PLoS One.

[29] Ramsbottom S.A., Molinari E., Srivastava S., Silberman F., Henry C., Alkanderi S. (2018). Targeted exon skipping of a CEP290 mutation rescues Joubert syndrome phenotypes *in vitro* and in a murine model. Proc. Natl. Acad. Sci. U. S. A..

[30] Li X., Yang S., Han L., Mao K., Yang S. (2020). Ciliary IFT80 is essential for intervertebral disc development and maintenance. FASEB J..

[31] Cogné B., Latypova X., Senaratne L.D.S., Martin L., Koboldt D.C., Kellaris G. (2020). Mutations in the kinesin-2 motor KIF3B cause an autosomal-dominant ciliopathy. Am. J. Hum. Genet..

[32] Hsu K.S., Chuang J.Z., Sung C.H. (2017). The biology of ciliary dynamics. Cold Spring Harb. Perspect. Biol..

[33] Keeling J., Tsiokas L., Maskey D. (2016). Cellular mechanisms of ciliary length control. Cells.

[34] Kim J., Lee J.E., Heynen-Genel S., Suyama E., Ono K., Lee K. (2010). Functional genomic screen for modulators of ciliogenesis and cilium length. Nature.

[35] Drummond M.L., Li M., Tarapore E., Nguyen T.T.L., Barouni B.J., Cruz S. (2018). Actin polymerization controls cilia-mediated signaling. J. Cell Biol..

[36] Rangel L., Bernabé-Rubio M., Fernández-Barrera J., Casares-Arias J., Millán J., Alonso M.A. (2019). Caveolin-1α regulates primary cilium length by controlling RhoA GTPase activity. Sci. Rep..

[37] Kim J., Jo H., Hong H., Kim M.H., Kim J.M., Lee J.K. (2015). Actin remodelling factors control ciliogenesis by regulating YAP/TAZ activity and vesicle trafficking. Nat. Commun..

[38] Bruck S., Huber T.B., Ingham R.J., Kim K., Niederstrasser H., Allen P.M. (2006). Identification of a novel inhibitory actin-capping protein binding motif in CD2-associated protein. J. Biol. Chem..

[39] Hernandez-Valladares M., Kim T., Kannan B., Tung A., Aguda A.H., Larsson M. (2010). Structural characterization of a capping protein interaction motif defines a family of actin filament regulators. Nat. Struct. Mol. Biol..

[40] Tang V.W., Brieher W.M. (2013). FSGS3/CD2AP is a barbed-end capping protein that stabilizes actin and strengthens adherens junctions. J. Cell Biol..

[41] Gaidos G., Soni S., Oswald D.J., Toselli P.A., Kirsch K.H. (2007). Structure and function analysis of the CMS/CIN85 protein family identifies actin-bundling properties and heterotypic-complex formation. J. Cell Sci..

[42] Rai A., Bleimling N., Vetter I.R., Goody R.S. (2020). The mechanism of activation of the actin binding protein EHBP1 by Rab8 family members. Nat. Commun..

[43] Wang P., Liu H., Wang Y., Liu O., Zhang J., Gleason A. (2016). RAB-10 promotes EHBP-1 bridging of filamentous actin and tubular recycling endosomes. PLoS Genet..

[44] Kim J., Krishnaswami S.R., Gleeson J.G. (2008). CEP290 interacts with the centriolar satellite component PCM-1 and is required for Rab8 localization to the primary cilium. Hum. Mol. Genet..

[45] Hsiao Y.C., Tong Z.J., Westfall J.E., Ault J.G., Page-McCaw P.S., Ferland R.J. (2009). Ahi1, whose human ortholog is mutated in Joubert syndrome, is required for Rab8a localization, ciliogenesis and vesicle trafficking. Hum. Mol. Genet..

[46] Baron Gaillard C.L., Pallesi-Pocachard E., Massey-Harroche D., Richard F., Arsanto J.P., Chauvin J.P. (2011). Hook2 is involved in the morphogenesis of the primary cilium. Mol. Biol. Cell.

[47] Coon B.G., Hernandez V., Madhivanan K., Mukherjee D., Hanna C.B., Ramirez I.B.-R. (2012). The lowe syndrome protein OCRL1 is involved in primary cilia assembly. Hum. Mol. Genet..

[48] Bachmann-Gagescu R., Dona M., Hetterschijt L., Tonnaer E., Peters T., de Vrieze E. (2015). The ciliopathy protein CC2D2A associates with NINL and functions in RAB8-MICAL3-regulated vesicle trafficking. PLoS Genet..

[49] Sobajima T., Yoshimura S., Maeda T., Miyata H., Miyoshi E., Harada A. (2018). The Rab11-binding protein RELCH/KIAA1468 controls intracellular cholesterol distribution. J. Cell Biol..

