# Supplementary material for: The Rab GTPase-binding protein EHBP1L1 and its interactors CD2AP/CIN85 negatively regulate the length of primary cilia via actin remodeling
Source: J Biol Chem. 2023 Feb 6;299(3):102985. doi: 10.1016/j.jbc.2023.102985 (PMC9986712; doi:10.1016/j.jbc.2023.102985)
Supplement: Supporting Figure S1 [file mmc1.pdf]

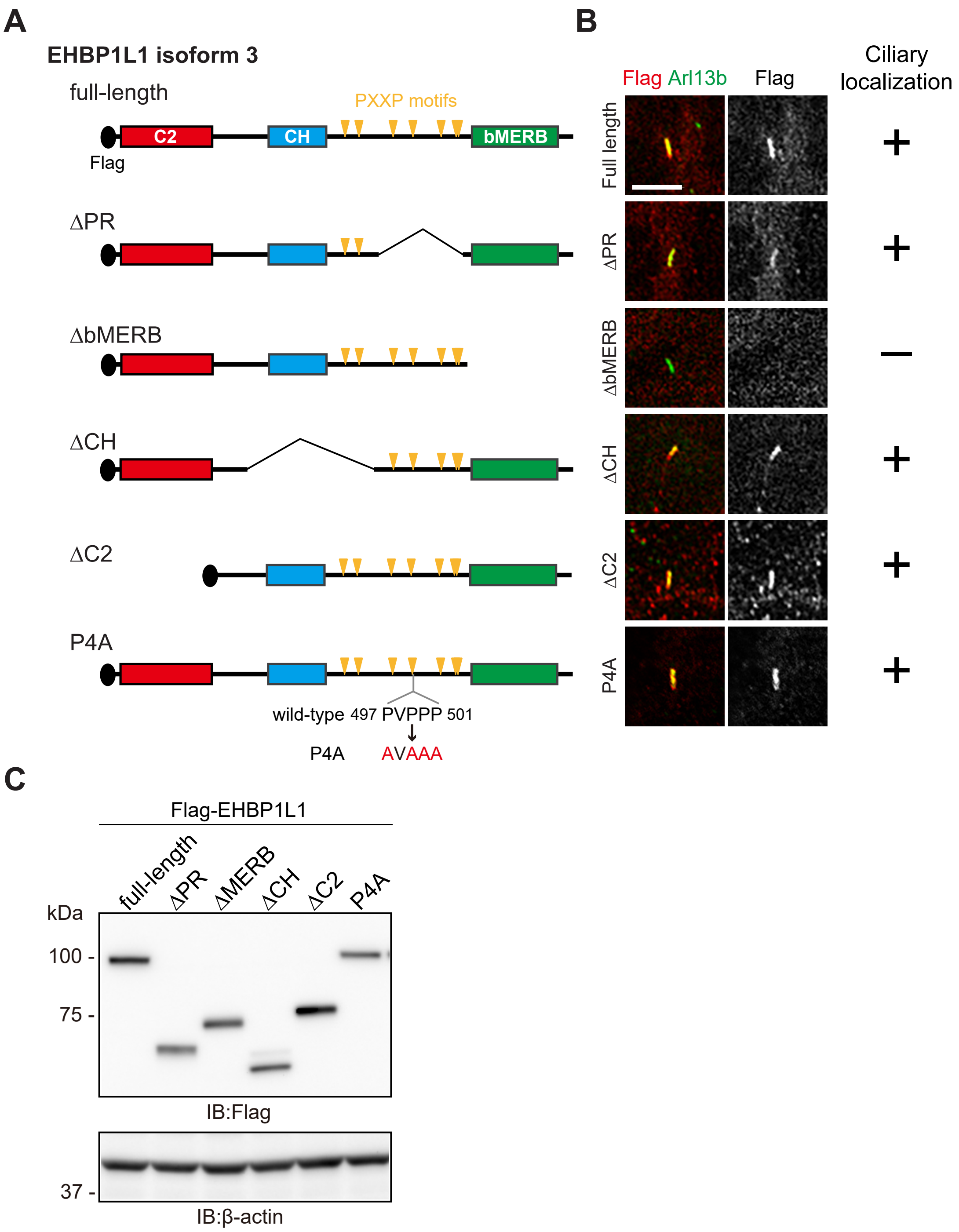

Figure S1 Expression and localization of EHBP1L1 and its mutant proteins in hTERT-RPE1 cells. *A*, Schematic representation of wild-type full length and mutants EHBP1L1. PR, proline-rich region; bMERB, bivalent Mical/EHBP Rab binding domain; CH, calponin homology domain; C2, Ca^{2+}/phospholipid-binding domain; P4A, full-length EHBP1L1 with four proline residues located at 497, and 499-501 replaced with alanine. *B*, Ciliary localization of EHBP1L1 and mutants. The Flag-tagged constructs were expressed in hTERT-RPE1 cells. The cells were then stained with Flag and Arl13b antibodies. The ciliary localization of EHBP1L1 and mutants were shown in the right. Only the ΔbMERB mutant failed to localize around the ciliary region, suggesting that Rab binding domain is important for ciliary localization. Scale bar: 5 μm. *C*, EHBP1L1 mutant expression was confirmed by immunoblotting with a Flag antibody and ß-actin was used as a loading control.
